# Editorial: Ovarian cancer-targeted medication: PARP inhibitors, anti-angiogenic drugs, immunotherapy, and more

**DOI:** 10.3389/fphar.2023.1222209

**Published:** 2023-06-09

**Authors:** Zhi-Bin Wang, Dan Liu, Guang Lei, Zhao-Qian Liu, Nayiyuan Wu, Jing Wang

**Affiliations:** ^1^ Hunan Key Laboratory of Cancer Metabolism, Hunan Cancer Hospital, The Affiliated Cancer Hospital of Xiangya School of Medicine, Central South University, Changsha, Hunan, China; ^2^ Public Service Platform of Tumor Organoids Technology, Hunan Gynecological Tumor Clinical Research Center, Changsha, Hunan, China; ^3^ Department of Gynecological Oncology, Tongji Hospital, Tongji Medical College, Huazhong University of Science and Technology, Wuhan, China; ^4^ Department of Experimental Radiation Oncology, The University of Texas MD Anderson Cancer Center, Houston, TX, United States; ^5^ Department of Clinical Pharmacology, Hunan Key Laboratory of Pharmacogenetics, National Clinical Research Center for Geriatric Disorders, Xiangya Hospital, Central South University, Changsha, Hunan, China; ^6^ Institute of Clinical Pharmacology, Engineering Research Center for applied Technology of Pharmacogenomics of Ministry of Education, Central South University, Changsha, Hunan, China

**Keywords:** ovarian cancer, targeted medication, immunomodulatory, drug resistance, PARP inhibitors, anti-angiogenic drugs

**FIGURE 1:**
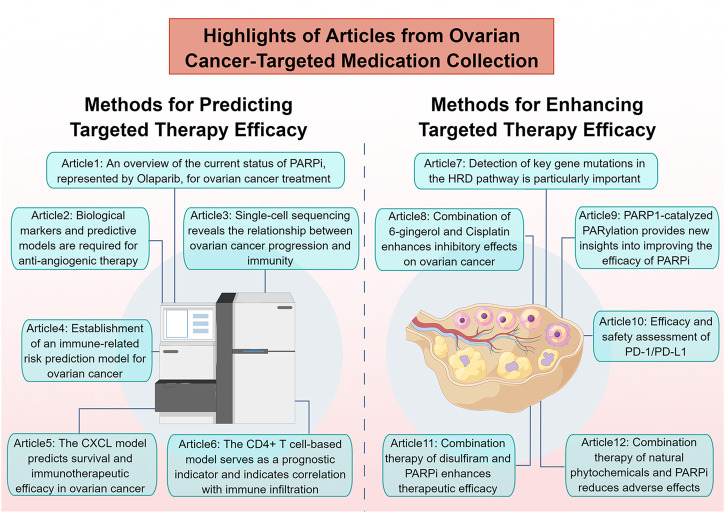
Highlights of articles from ovarian cancer-targeted medication collection (By FigDraw).

Ovarian cancer (OC) is a highly fatal malignancy, with tumor reduction and platinum-based chemotherapy being its primary treatments. However, acquired platinum resistance poses a challenge to patient management. Targeted therapies, such as PARP inhibitors (PARPi) and anti-angiogenic agents, are replacing conventional chemotherapy. Next-generation sequencing offers promise in identifying specific molecular targets for personalized treatment. Immunotherapy and modulation of the ferroptosis pathways also present new avenues for targeted therapy. Improving the efficacy and reducing side effects of existing therapies and exploring new options are pressing challenges. This Research Topic covers Research Topic in pharmacology, including immune-targeted therapy, prognostic biomarkers, and single-cell sequencing analysis of the immune microenvironment in OC (Figure 1). The following section provides a concise summary of the major highlights from the twelve articles included in this Research Topic.

1. Olaparib, a PARPi, has reshaped the treatment scenario of metastatic OC as a maintenance therapy post-platinum-based chemotherapy. Maiorano et al. review summarizes the efficacy and safety of several clinical trials, including Study 42, Study 19, SOLO2, OPINION, SOLO1, and PAOLA-1, which led to the FDA and EMA approval of olaparib for maintenance treatment in women with high-grade epithelial ovarian, fallopian tube, or primary peritoneal cancer without platinum progression, in the platinum-sensitive recurrent OC, and in newly diagnosed cases with BRCA mutations. Additionally, the review discusses the future developments and potential applications of olaparib in OC treatment.

2. Although anti-angiogenic agents have been developed to target tumor angiogenesis in OC, Mei et al. review emphasizes the need for biomarkers and predictive models to guide precision therapy. The review aims to provide an updated understanding of the mechanisms of tumor angiogenesis and the latest reports on the clinical trial outcomes and resistance mechanisms of anti-angiogenic agents in OC.

3. Zhang et al. study provides key insights into the differences between healthy ovarian and ovarian cancer cells and demonstrates the potential of ScRNA-seq analysis in guiding personalized treatments. Furthermore, the study finds variations in immune-related and apoptosis-related gene expression between healthy ovarian and ovarian cancer cells. These findings provide key insights for further research into the treatment of OC. Overall, ScRNA-seq analysis has the potential to improve our understanding of OC at the cellular level and guide personalized treatments for patients.

4. Wu et al. study highlights the potential of a machine learning-based immune-related risk model to improve prognostic prediction and guide personalized treatment for HGSOC. The developed TMErisk scoring system demonstrated superior performance in predicting HGSOC prognosis across cohorts and was associated with BRCA1 mutation, C-X-C motif chemokine ligands deletion, and carcinogenic activation pathways. The low TMErisk group exhibited favorable prognosis and better immune response. Overall, this study offers valuable insights into the diversity of cell components in the TME of HGSOC and guides the development of potential therapeutic techniques for addressing tumor immunosuppression and enhancing the response to cancer therapy.

5. The main conclusion of Li et al. study is that the CXCL score has the potential to be a reliable biomarker for predicting clinical outcomes and immunotherapy responses in individual OC patients. The CXCL scoring model showed effectiveness in predicting immunotherapy response by assessing tumor microenvironment cell infiltration, tumor mutational burden estimation, PD-L1/CTLA4 expression, and immunophenoscore analysis. These findings suggest that the CXCL score has the potential to guide personalized immunotherapy in OC patients and improve treatment outcomes.

6. Hua et al. study offers valuable insights into the molecular mechanisms and clinical management of OC and may guide personalized treatments for patients. A risk signature consisting of 11 CD4^+^ conventional T-cells-related genes (CD4TGs) has prognostic value in OC and can guide clinical management. The study finds that a high risk score is significantly associated with poorer prognosis and could be used as an independent prognostic biomarker. The low-risk group patients tended to exhibit higher immune infiltration, immune-related gene expression, and greater sensitivity to immunotherapy and chemotherapy. These findings provide valuable insights into the molecular mechanisms and clinical management of OC and may guide personalized treatments for patients.

7. Huang et al. review highlights the potential of molecular targeted therapies to improve treatment outcomes for OC patients by overcoming the limitations of traditional chemotherapy. Specifically, the review highlights the importance of detecting genetic mutations such as BRCA mutations and mutations of other homologous recombination repair defect (HRD) genes, which can guide the targeted drug treatment of patients.

8. The study conducted by Salari et al. compares the effects of cisplatin, 6-gingerol, and their combination on OC cells *in vitro* and *in vivo*. The results show that the combination therapy significantly promoted apoptosis and induced a higher S sequence extent in the cell cycle. Moreover, the expression of genes associated with apoptosis was amplified by the combination therapy, while the expression of genes linked to angiogenesis decreased. Therefore, this study highlights the potential benefits of using complementary treatments and herbal medicines to improve the performance of conventional medicine in treating OC.

9. According to Zhu et al. review, while PARPi are commonly used to treat BRCA1/2-deficient breast and ovarian cancers, resistance to PARPi frequently occurs. Therefore, the review focuses on identifying novel substrates and regulators of poly (ADP-ribosyl)ation (PARylation), which is catalyzed by PARP1, as potential targets for therapeutic intervention to overcome PARPi resistance. This study highlights the importance of understanding the underlying mechanisms of PARP1-catalyzed PARylation and provides new insights into potential strategies for improving the efficacy of PARP inhibitors in treating breast and ovarian cancers.

10. From the perspective of Zeng et al. study, while PD-1/PD-L1 inhibitors hold potential as a targeted therapy for recurrent/refractory OC, they offer modest efficacy when used alone. Their meta-analysis of 11 studies with 990 patients demonstrates an ORR of 6.7%, DCR of 37.9%, median OS of 10.70 months, and median PFS of 2.24 months, as well as TRAEs of 70.9% and iAEs of 29%. Therefore, the study suggests caution in using PD-1/PD-L1 inhibitors and the need for further research to optimize their use in treating OC.

11. Based on their research, Tang et al. and others conclude that disulfiram, in combination with PARPi, has potential as a therapeutic candidate for OC treatment. The combination significantly decreased the viability of OC cells and increased the expression of DNA damage index gH2AX and PARP cleavage. Based on these findings, the study offers a novel treatment strategy for patients with OC.

12. The principal outcome of Wang et al. study indicates that natural phytochemicals, including sulforaphane, lycopene, catechin, and curcumin, exhibit potential efficacy in mitigating and treating the negative effects correlated with BRCA mutations and PARPi exposure among OC patients. These bioactive substances demonstrate significant therapeutic efficacy against atherosclerosis, nausea, and vomiting, which are prevalent chemotherapy-related side effects. The author anticipates that these findings may offer novel perspectives for exploring innovative and effective therapeutic approaches for treating BRCA-mutated OC patients.

In summary, this Research Topic provides insights into the development of targeted therapies for OC and offers solid evidence to improve their efficacy and reduce toxicity. However, due to the histological characteristics of the ovarian tissue microenvironment and its nature as a “cold tumor,” research on targeted therapies for OC, represented by immunotherapy, still faces challenges. However, although the path may be long, progress will ultimately be made.

